# Legg–Calvé–Perthes disease overview

**DOI:** 10.1186/s13023-022-02275-z

**Published:** 2022-03-15

**Authors:** Armando O. Rodríguez-Olivas, Edgar Hernández-Zamora, Elba Reyes-Maldonado

**Affiliations:** 1 Department of Morphology, Escuela Nacional de Ciencias Biológicas, Instituto Politécnico Nacional, Prolongación de Carpio y Plan de Ayala s/n, Col. Santo Tomás, Miguel Hidalgo, C.P. 11340 Mexico City, Mexico; 2 Genomic Medicine, Instituto Nacional de Rehabilitación Luis Guillermo Ibarra Ibarra, Mexico City, Mexico

**Keywords:** LCPD, Diagnosis, Treatment, Environmental factors, Biochemical factors, Genetic factors

## Abstract

**Background:**

Legg–Calvé–Perthes Disease (LCPD) is a necrosis of the femoral head which affects the range of motion of the hips. Its incidence is variable, ranging from 0.4/100,000 to 29.0/ 100,000 children. Although LCPD was first described in the beginning of the past century, limited is known about its etiology. Our objective is to describe the main areas of interest in Legg–Calve–Perthes disease.

**Methods:**

A review of the literature regarding LCPD etiology was performed, considering the following inclusion criteria: Studies reporting clinical or preclinical results. The research group carried out a filtered search on the PubMed and Science Direct databases. To maximize the suitability of the search results, we combined the terms ‘‘Perthes disease” OR “LCPD” OR “children avascular femoral head necrosis” with “diagnostic” OR “treatment” OR “etiology” as either key words or MeSH terms.

**Results:**

In this article been described some areas of interest in LCPD, we include topics such as: history, incidence, pathogenesis, diagnosis, treatment and possible etiology, since LCPD has an unknown etiology.

**Conclusions:**

This review suggests that LCPD has a multifactorial etiology where environmental, metabolic and genetic agents could be involved.

## Background

Legg–Calvé–Perthes Disease (LCPD) is characterized by, unilateral or bilateral, necrosis of the femoral head (FH). Which affects the range of motion of the hip. In our experience, patients generally report pain in the affected joint, which intensifies during and after physical activity. On the other hand, lameness or trendelenburg gait is characterized by being the main sign for which they come for consultation. Its incidence is variable, ranging from 0.4/100,000 to 29.0/ 100,000 < 15 years old children [1, 2].

Although LCPD was first described in the beginning of the past century and has been studied for more than 100 years, limited is known about its etiology [3]. Bone remains with distinctive alterations related to LCPD have been found in Argentina, the Czech Republic, Italy and China, suggesting this disease has been present from very remote times [4–7]. Between 1909 and 1910, radiological advances allowed differentiating LCPD from other pathologies like fractures, rickets, septic arthritis, and tuberculous arthritis. Thus, LCPD was described almost simultaneously in different countries; by Arthur Legg, Jacques Calvé, Georg Perthes and Henning Waldenström independently [8–10]. In 1922, Waldeström proposed to classify the disease in four stages: osteonecrosis stage, fragmentation stage, reossification stage, and healed stage. This classification is still useful today [8, 11]. However, even though different diagnosis methods and treatments have been used throughout history, the etiology of LCPD remains unknown. Nonetheless, there are several theories proposing environmental, metabolic and genetic factors as causative agents of the disease [3].

## Methods

**Fig. 1:**
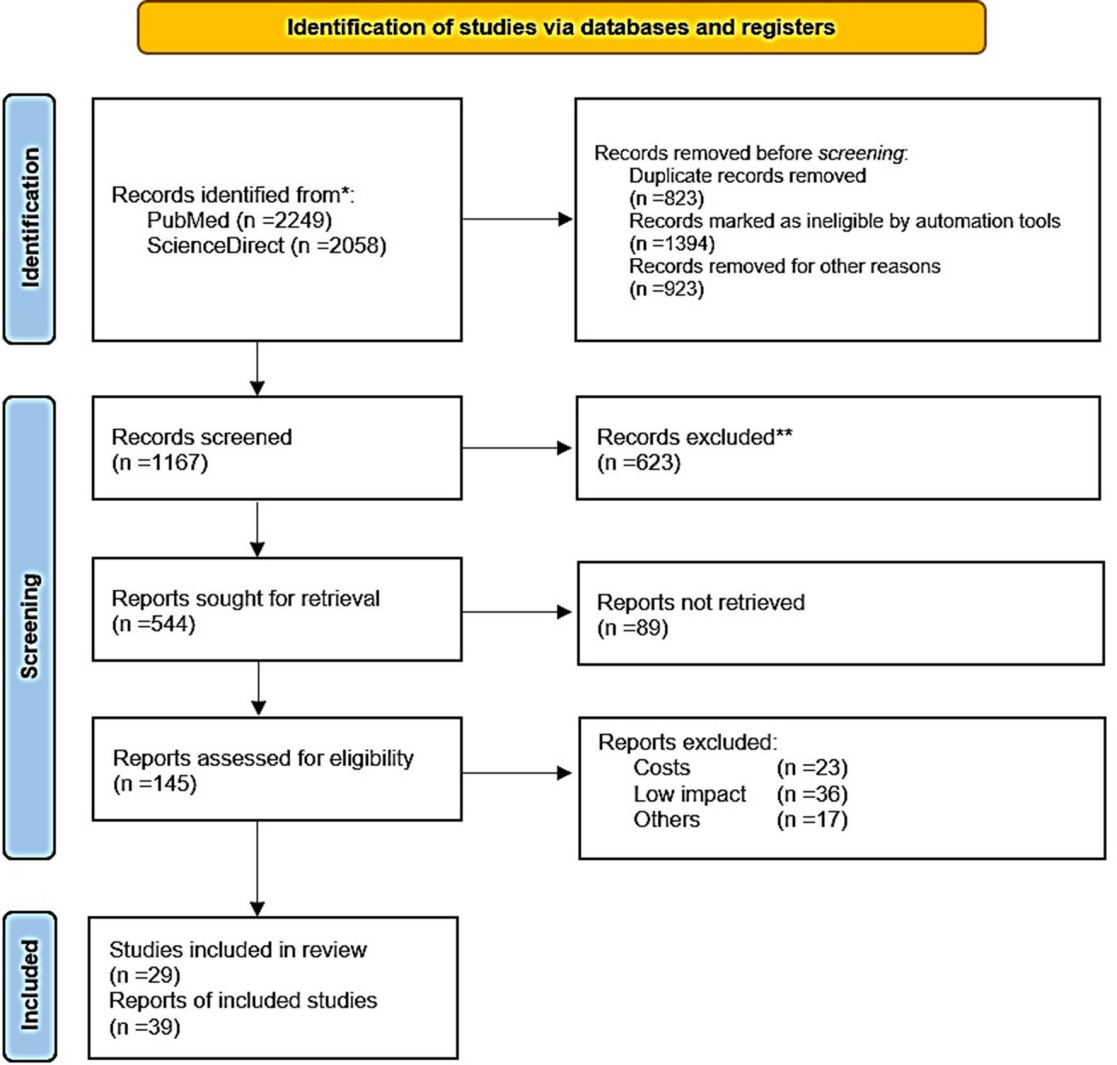
Flow diagram

A review of the literature regarding LCPD etiology was performed, considering the following inclusion criterions: Studies reporting clinical or preclinical results. Dealing with the etiology or pathogenesis of LCPD, regardless of the level of evidence. In addition, due to the limited information written about this topic, it was decided to include all those articles with less than 25 years of having been accepted and / or published. The research group carried out a filtered search on the PubMed, from their date of inception to October 1st 2021 (Fig. 1). To maximize the suitability of the search results, we combined the terms ‘‘Perthes disease” OR “LCPD” OR “children avascular femoral head necrosis” with “diagnostic” OR “treatment” OR “etiology” as either key words or MeSH terms.

### Epidemiology

The incidence of LCPD varies widely among countries, cities and races ranging from 0.4/100,000 to 29.0/100,000 children. LCPD usually appears from the age of 3 to 12 years old, with the highest rate of occurrence at the age of 5 to 7 years old. Boys are affected three to five times as often as girls, and the disorder is bilateral in 10–24% of patients, with a correlation to inheritance in approximately 8–12% of patients [2, 12, 13].

### Pathogenesis

The pathogenesis of LCPD is complex. From a mechanical point of view, deformities in the FH will occur when the forces applied to the FH are greater than its capacity to resist deformation. Animal models have shown that necrosis decreases the mechanical and supportive properties of the FH, the articular cartilage, and the bone. It is suggested that the mechanical properties of infarcted bones are compromised as a result of various mechanisms taking place during different stages of the disease [14].

First, in the avascular stage, the calcium increase in the necrotic bone makes it more prone to microdamage, which compromises the mechanical properties of the FH. Necrosis will lead to a decay of bone cells, osteoclasts, and osteoblasts, causing microfractures to remain unrecognized and/or unrepaired. Then, during the revascularization stage, the necrotic bone will be resorbed, further compromising mechanical properties. The hip is one of the main load-bearing joints, so it is important to consider the forces applied to the joint, as they will influence the degree of deformity in the FH [14, 15].

From the radiological point of view, the process of ischemia and subsequent bone regeneration have been divided into several stages (Table 1). The identification of the phase is of utmost therapeutic importance.

**Table 1:** LCPD stages

Stage	Characteristics
Initial or necrosis phase	Interruption of vascular supply and bone necrosis, at this stage the FH is very vulnerable to the forces acting on it; radiologically the Waldenström sign is visible, which is characterized by increased joint space, secondary to a subchondral fracture, this is the earliest radiological sign
Fragmentation phase	It is initiated by a process of resorption of necrotic bone, radiologically dense bone islets appear, the central ones are condensed, and the lateral ones undergo osteolysis producing an image with multiple lines
Reossification phase	The density is displaced in the opposite direction, the epiphysis is invaded by vessels, the dense islets are reabsorbed and irregular bone tissue is formed, which then trabeculates, and repair begins with disappearance of the metaphyseal osteolysis
Final phase, of healing or residual deformity	The necrotic bone is completely replaced by newly formed bone. The newly formed bone has a lower rigidity so it can be remodeled in such a way that the morphology of the FH adapts to the shape of the insertion hole or not, this process will not be definitive until the end of bone maturation. The result may be a deformed FH

This table includes information from the following references

Kim HK, Herring JA. Pathophysiology, classifications, and natural history of Perthes disease. Orthop Clin North Am 2011;42(3):285–95

Wenger DR, Pandya NK. A brief history of Legg–Calvé–Perthes disease. J Pediatr Orthop 2011;31(2)130–136

Dustmann HO. [Etiology and pathogenesis of epiphyseal necrosis in childhood as exemplified with the hip]. Z Orthop Ihre Grenzgeb. 1996 Sep-Oct;134(5):407–12

The duration of each stage is very variable, but, in general, the necrosis and fragmentation stage last about six months; the reossification stage, from 18 months to three years; and the final phase, until bone maturation. According to other authors, the fragmentation phase lasts approximately one year, and the reossification phase, from three to five years [15, 16].

### Diagnosis

**Fig. 2:**
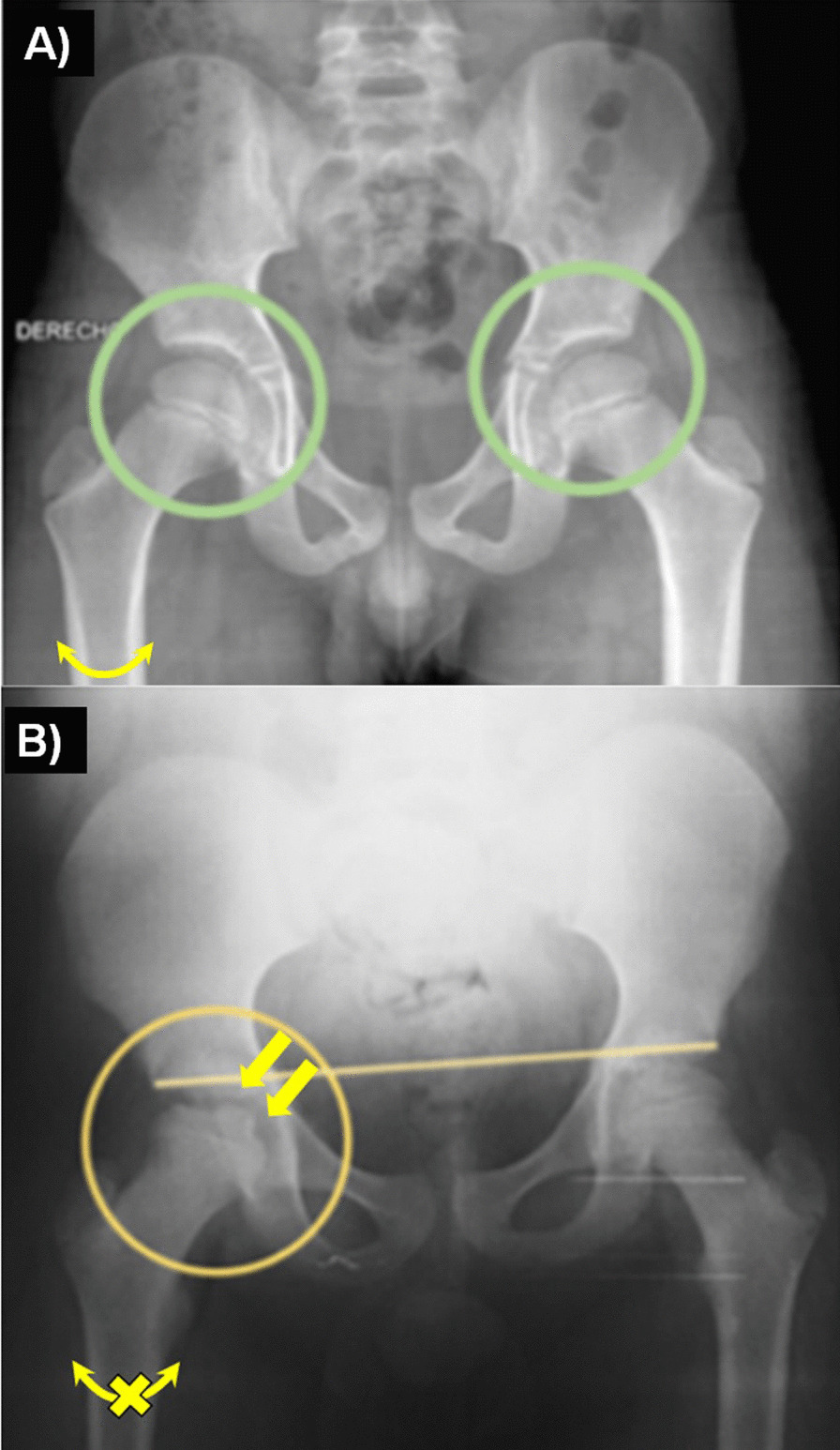
AP radiography. In the AP X-ray the deformity of the hip and femoral head characteristic of LCPD is demonstrable, **A** healthy control, **B** LCPD patient. Courtesy of INR-LGII genetics laboratory 2017

Due to lack of information, LCPD diagnosis can be difficult; nevertheless, there are some important diagnostic criteria (Table 2). Differential diagnoses that must be considered given the radiographic findings include, coxitis fugax, Meyer dysplasia, epiphyseal dysplasia, spondyloepiphyseal dysplasia, chondroblastoma, juvenile idiopathic arthritis, drug-induced femoral head necrosis, Gaucher’s disease, sickle cell anemia, thalassemia, achondroplasia and Klinefelter syndrome [17–19] (Fig. 2).

**Table 2:** Diagnostic criteria

Clinical features	Pain is primarily localized in the hip, occasionally accompanied by leg and knee pain, most of the patients shows limited hip internal rotation. Patients often have a history of practice of high impact sports, smoke exposure, and deprivation
X-ray imaging	Anteroposterior and frog-leg positioning are the basic X-ray positions used for diagnosis of ONFH, and the X-ray manifestations are typically osteosclerosis, cystic change, and a “crescent sign” in earlier stages. After collapse, there is a loss of sphericity of the femoral head and degenerative arthritis in the late stages (Fig. 2A, B)
Magnetic resonance imaging	MRI seems to be the best method, MRI may show proximal femoral abnormalities before radiography in the setting of Legg–Calvé–Perthes disease, allowing appropriate diagnosis and prompt treatment. MRI can also assess for revascularization, healing, and multiple complications. MRI examination has a high sensitivity for ONFH, demonstrated as a limited subchondral linear-shaped low signal intensity in T1-weighted images (T1WIs) or a “double-line sign” in T2-weighted images (T2WIs)
Computed tomography scanning	Computed tomography (CT) scanning usually reveals zones of osteosclerosis surrounding the necrotic bone and repaired bone or shows subchondral bone fracture

### Classification

In order to predict the prognosis and decide on the appropriate treatment, there are classifications that mostly consider the region and area affected.

**Fig. 3:**
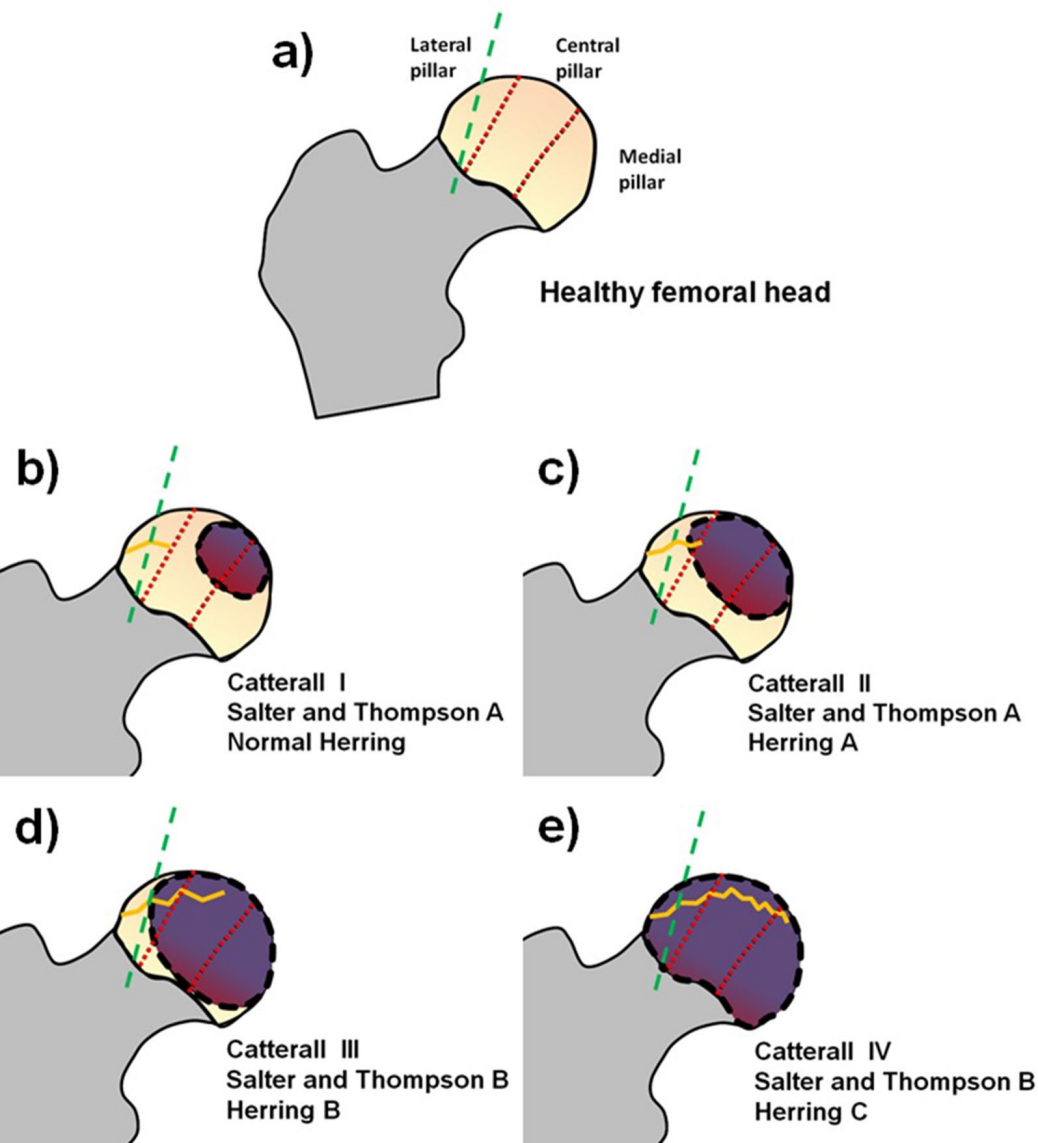
Dotted lines divide the femoral head into medial, central and lateral pillars. The gray dashed line indicates approximately the middle of the lateral pillar. Its dashed outline is the necrosed area. The zig-zag line represents the subchondral fracture size (fs). **a** Healthy femoral head. **b** Cavity with 25% of total area lost; its discontinuous outline is a necrosed area and there is a subchondral fissure (fs). **c** Loss of ~ 50% of total area, increased necrosed area, increased size of fs and loss of lateral abutment height of < 50%. **d** Loss of > 50% of total area, increased fs and loss of lateral abutment height of ~ 50%. **d** Total cavity with loss of nearly 100% of area, maximum subchondral damage and damage of > 50% of lateral abutment

Catterall, in 1971, divided the disease into four grades, according to the extent of the epiphyseal lesion—Type I: 0–25%; Type II: 25–50%; Type III: > 50%; and Type IV: 100% (Fig. 3).

In 1984, Salter and Thompson described a classification of two groups (A and B), which were defined by the extent of subchondral fracture visible on axial radiographs in the early stage of the disease. The disadvantage of this one, is that not all patients are diagnosed at an early stage (Fig. 3) [20, 21].

The most recent classification was proposed by Herring in 1992. It is based on the height of the lateral pillar of the FH epiphysis in the fragmentation period of the disease and is divided into three groups—group A: there is no involvement of the lateral pillar, and its full height is maintained; group B: height loss < 50%; and group C: height loss > 50%. The predictive value of the Herring classification is higher in the early stages of the disease. Recently, a fourth group has been proposed between groups B and C, in which the lateral abutment is narrow, poorly ossified or maintains 50% of its height (Fig. 3) [22, 23].

### Treatment

**Fig. 4:**
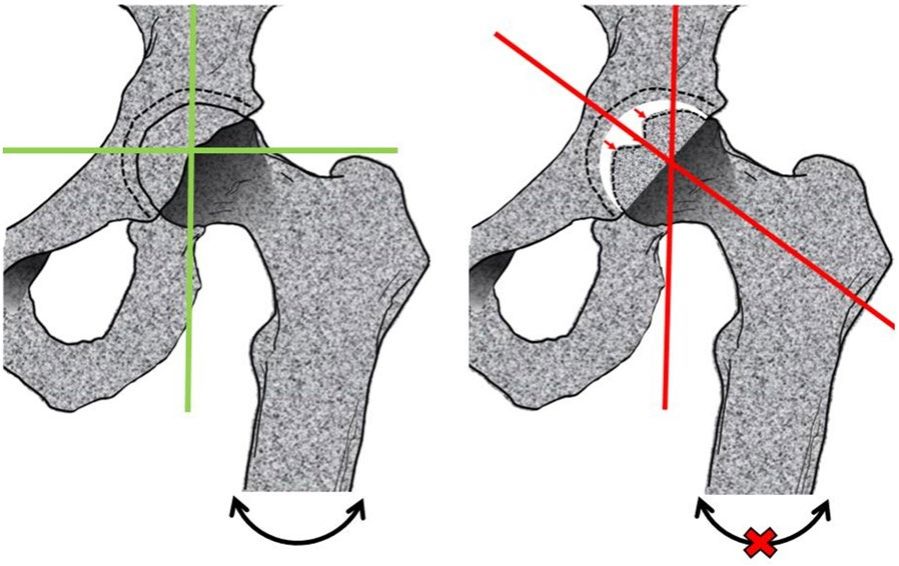
Loss of the hip junction axis. The deformity in the femoral head, as well as the shortening of the affected extremity will cause the loss of the hip junction axis, due to mechanical damage, causing the characteristic symptoms of LCPD

The main symptoms of LCPD are lameness and localized pain in the hip, radiating to the thigh and knee; nonetheless, some cases present painless limp. It is common to find limitation of abduction and internal rotation, as well as limitation in flexion of approximately 20 degrees. In some cases, there is shortening of the affected extremity [24–26]. Most of these symptoms are linked to the loss of the hip joint axis (Fig. 4).

Since the degree of FH deformity varies widely among patients, treatment is decided on a case-by-case basis. Existing treatments range from follow-up and observation to surgical procedures on the femur and hip. All treatments are aimed at preventing deformity of the FH, incongruence of the affected hip, and early onset of coxarthrosis [27].

The choice of treatment is made based on the radiographic characteristics of the patient. In general, surgical treatment is not necessary in patients at early stages, which have full and painless range of motion of the hip and are low risk radiologically regarding the FH, such as in Catterall’s grades I or II and in Salter and Thompson’s group A.

Patients with hip pain or stiffness should rest for 5 to 7 days, and nonsteroidal anti-inflammatory drugs may be prescribed to minimize some symptoms. Patients who do not respond to rest are hospitalized and placed in bilateral skin traction with progressive abduction during 7 to 10 days to immobilize the joint. Joints that do not regain motion are subjected to arthrography. It should be noted that, if the deformity of the FH prevents hip abduction, some other treatment needs to be considered. Percutaneous hip abductor release can be used in joints that show a contracture on abduction but still retain their shape. Once maximum hip motion is obtained, containment therapy is considered for radiological groups with poor prognosis, but the efficacy of such treatments is variable depending on the case treated [27–29].

**Fig. 5:**
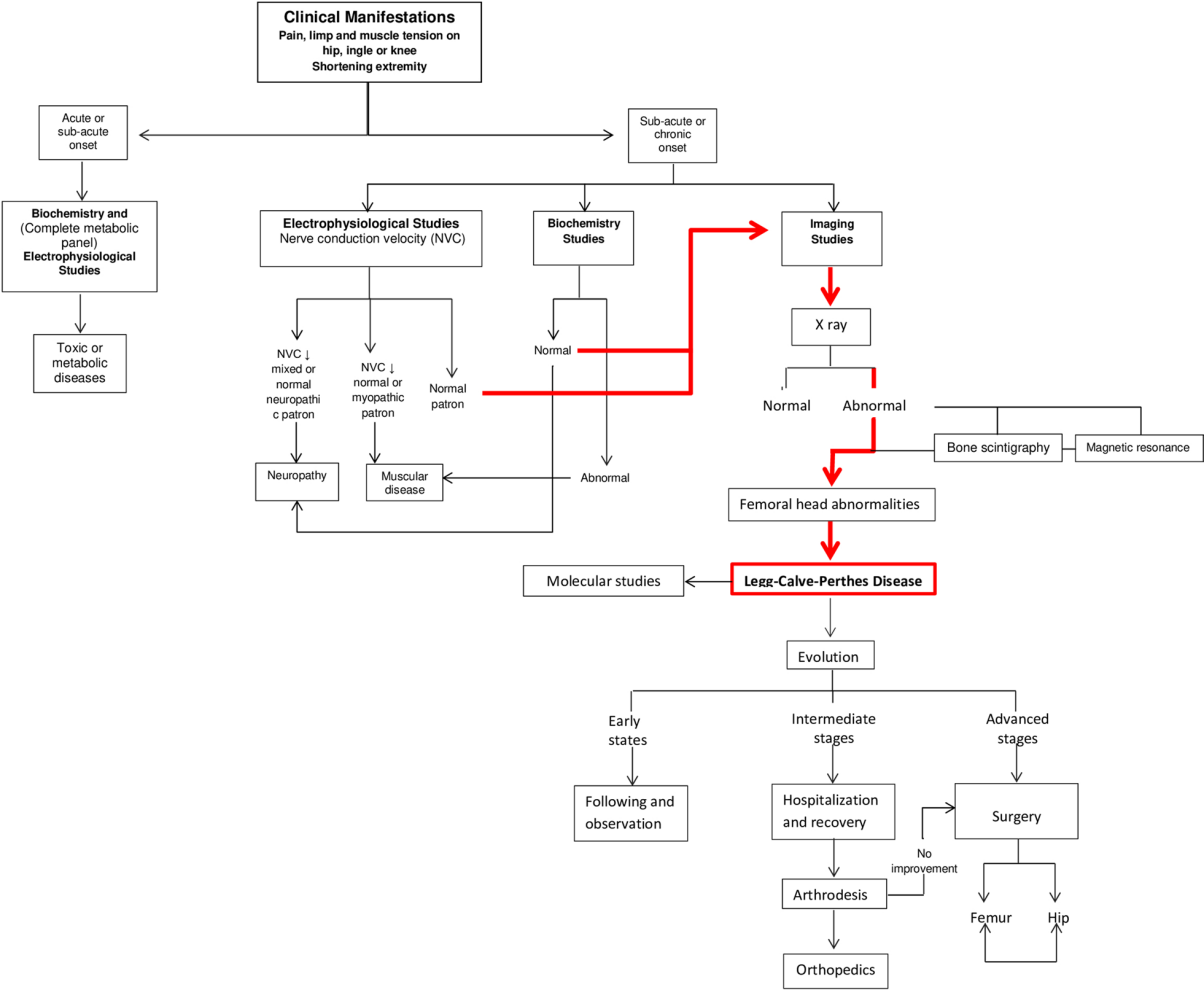
Study algorithm for patients with Legg–Calvé–Perthes disease

Patients with a greater area of damage in the FH may be candidates for surgical treatments, such as innominate osteotomy in the pelvis or osteotomy in the rod of the hip, in order to maintain the femoral head as congruent as possible with the pelvis. Shelf arthroplasty has been recommended for children older than 8 years or in Catterall groups III and IV. Patients with non-containable hips and active disease or those with recovered hips who have painful hinge abduction may be candidates for hip abduction-extension osteotomy. Skeletally mature patients can be treated by cheilectomy [28, 30]. Femoral osteotomy is more commonly used than pelvic osteotomy; however, pelvic osteotomy is considerably more commonly used in North America, Australia and South America, while femoral osteotomy is more often performed in Europe, Asia and Africa [31]. However, our experience with LCPD suggested a different diagnostic algorithm (Fig. 5).

### Prognosis

The sequelae found in LCPD patients after the age of 40 are minimal, and the long-term prognosis tends to be good in 60% to 80% of the total cases. There are multiple prognostic factors in LCPD, such as age of onset and diagnosis, sex, range of motion of the hip, and classification. While, in general, patients between five to seven years old have a better prognosis than those of older ages, patients in adolescence have a poor prognosis. In the case of women, it is reported that they tend to have a worse prognosis; however, Guille et al*.*, report that the prognosis can be similar between men and women. Finally, bilateral cases also present a poor prognosis. The severity of the damage, the classification of the patient, the size of the damaged area and the treatment used must be considered [32–34].

### Etiology

The cause of LCPD is unknown. Different etiologies have been proposed; nevertheless, LCPD may be caused by multiple etiologic factors that share a common final pathogenic pathway. A new perspective is that LCPD is a multifactorial disease caused by a combination of environmental, metabolic and genetic factors. However, during the diagnosis, different etiological factors could be considered.

### Environmental, metabolic and genetics factors described on the LCPD

Regarding race (Table 3), LCPD occurs with a higher incidence in Caucasians, with the number of cases declining in Asian people and diminishing even more in black people. The incidence of LCPD varies widely in different geographic regions. On the other hand, sex could be considered as another factor, since LCPD mainly affects men (Table 3). Some studies describe a link between latitude and the incidence of the disease, proposing that climate, hours of sun exposure, among other factors, could be triggers of LCPD [14]. Other studies indicate that, in lower socioeconomic levels (Table 3), there is an increase in the incidence of LCPD, so it is presumable that the nutritional status of the patient could be involved in the development of the disease [35, 36].

**Table 3:** Etiological factors related to Legg–Calve–Perthes disease

Factor	Year	References
Environmental		
Deprivation	2000	Kealey et al. J Bone Joint Surg Br
Urbanisation	2000	Kealey et. al. J Bone Joint Surg Br
Race	2012	Perry DC et al. Am J Epidemiol
Gender	2012	Perry DC et al. Am J Epidemiol
Somke exposure	2017	Perry DC et al. Bone Joint J
Metabolic		
Tissue-plasminogen activator	1996	C J Glueck et al. Bone Joint Surg Am
Resistance to activated protein C	1997	C J Glueck et al. Clin Orthop Relat Res
Abnormalities in factor V	2004	Balasa VV. et al. J Bone Joint Surg Am
Anticardiolipin antibodys	2004	Balasa VV. et al. J Bone Joint Surg Am
Low antithrombin activity	2005	Yilmaz D. et al. Pediatr Int
High levels of soluble Thrombomodulin	2008	Aksoy M. et al. Hematology
Hight levels of Factor VIII	2010	Vosmaer A et al. Bone Joint Surg Am
Protein S deficiency	2010	Vosmaer A et al. Bone Joint Surg Am
Increased Selectin E	2014	Ismayilov V et al. J Pediatr Hematol Oncol
Increased Selectin A	2014	Ismayilov V et al. J Pediatr Hematol Oncol
Gens/polymorphism		
Receptor Activator of Nuclear factor Kappa-B (RANK) rs3018362. 18q21.33. OMIM *603499	1997	Anderson DM et al. Nature
Osteoprotegerin (OPG) rs2073618. 18q24.12. OMIM *602,643	1998	Morinaga T et al. Biochem Biophys Res Commun
Type I collagen (COL1A1) rs1107946. 17q21.33. OMIM 120150	1999	Sainz J et al. J Clin Endocr Metab
RANK Ligand (RANKL) rs12585014. 13q14.11. OMIM *602642	2004	Koga T et al. Nature
Type II collagen (COL2A1) rs121912891, 12q13.11. OMIM 120140	2014	Li N, et al. Plos one
Toll-like receptor 4 (TLR-4) rs4986790. 9q33.1. OMIM 603030	2016	Adapala NS, et al. Am J Pathol
Tumor necrosis factor α (TNF-α) rs1800629. 6p21.33. OMIM 191160	2018	Azarpira MR, et al. J Orthop
Interleukin 6 (IL-6) rs 1800795. 7p15.3. OMIM 147620	2021	Akbarian-Bafghi MJ, et al. Fetal Pediatr Pathol
Nitric oxide synthase (eNOS) rs1799983. 7q35-36. OMIM 163729	2019	Azarpira MR, et al. J Orthop

*rs* RefSnp, reference single nucleotide polymorphism, *OMIM* Online Mendelian Inheritance in Man

Some studies have shown that there is a correlation between LCPD and exposure to tobacco and wood smoke (Table 3) [37, 38]. It has been found that there is a slight correlation between LCPD, growth disturbances and low birth weight [3, 39]. Neal et al*.*, report that there is a high rate of obesity in patients with LCPD, since it involves several risk factors such as poor nutrition, inflammation and increased mechanical load [40]. Mechanical overload seems to predispose to LCPD, as it has been shown that repetitive gymnastics training and errors in technical action can have a significant effect on the development of vascular necrosis in the FH, leading many gymnastics athletes to suffer from LCPD [41]. Animal models showed that, after overloading the joint, changes in the FH similar to those found in LCPD are noticeable [42]. Another important aspect is that in children with attention deficit hyperactivity disorder, who tend to be more active, there is an increased risk of LCPD [43].

Biochemical alterations affect bone development through multiple factors: obesity, abdominal circumference, high density lipoproteins (HDL), tumor necrosis factor alpha (TNF-α), interleukins (IL-1β and IL-6), and defects in lipid metabolism. These have effects on bone metabolism and are therefore considered risk factors for osteonecrosis and LCPD [40, 44, 45]. High concentrations of leptin and lipoprotein A, proteins highly influenced by obesity, have been found in serum of patients with LCPD [45, 46].

The disease is commonly linked to alterations to thrombophilia or hypercoagulable states (Table 3), such as factor V Leiden mutation, overactivity of FVIII and prothrombin, alterations in natural anticoagulants like protein C and S, hypofibrinolysis and increased selectins (E and P); however, data also suggests that inflammation and endothelium could be important factors in the development of LCPD [47–50].

Inflammation has an impact on bone modeling; in fact, it has been proposed that heterozygotes of the IL-6 G-174C/G-597 mutation are more likely to develop LCPD [51]. Kamiya et al*.*, observed that there is increased IL-6 in the synovial fluid of patients with LCPD. The neutrophil/lymphocyte ratio is a marker of subclinical inflammation, which increases as the damage in the FH enlarges; it could be considered, then, that inflammation has implications in the appearance of LCPD and its severity [52, 53].

Avascularity plays a key role in the etiology of LCPD. Risk factors such as hypertension, increased lipoprotein A, abnormalities in vascular architecture, decreased gill diameter, and decreased blood flow velocity have been described in patients with LCPD, suggesting that the cardiovascular apparatus may be compromised in multiple ways [54, 55]. Some authors have reported mutations associated with different metaphyseal changes of the FH that alter the structure of type II collagen. These alterations cause a localized collapse in the matrix surrounding the blood vessels, which promotes avascularity during development when there are early signs of osteonecrosis [56, 57].

There are studies that describe families with more than one affected member, which evidences genetic mechanisms may be involved in LCPD, and inheritance patterns, from autosomal recessive to polygenic, have been proposed. However, in families with a high rate of affected individuals, there appears to be an autosomal dominant mode of inheritance [57, 58]. Loder et al*.*, found that the rate of occurrence of LCPD in first, second, and third-degree relatives combined was 1:39, and, among siblings, 1:26; i.e., 35 and 50 times more respectively than in the general population [2]. LCPD has been linked to different genetic disorders such as Alagille syndrome, Albright hereditary osteodystrophy [59, 60] and trichorhinophalangeal syndrome, which are characterized by craniofacial anomalies and skeletal abnormalities of variable degree [61]. Epigenetic changes could be involved in the onset of LCPD, as Zheng et al*.*, report that there are lower levels of methylation in patients with LCPD, affecting bone and cartilage development in multiple ways [62].

Since hypercoagulable states could be related to the origin of LCPD, there are studies that relate mutation in factor V Leiden, polymorphisms in prothrombin (PT), and methylenetetrahydrofolate reductase (MTHFR) with the risk of developing LCPD (Table 3) [63, 64].

Inflammation is also considered important in the development of LCPD. Azarpira et al*.*, reported that the endothelial nitric oxide synthase (eNOS) polymorphisms 894G > T and -786 T > C increase the risk of suffering from LCPD (Table 3), and that the polymorphisms TNF-α -308G > A and TNF-α -238C > T could not be directly related to LCPD, but could be related to the development of osteonecrosis of the FH (Table 3) [65, 66]. Furthermore, the eNOS is involved in numerous physiological processes, including angiogenesis, thrombosis, coagulation, and fibrinolysis, and, recently, the G894T mutation in the eNOS gene was described as a risk factor in LCPD (Table 3) [67].

## Discussion

Despite its low incidence, LCPD represents a major global health problem, since it affects a significant part of the world population [2, 12, 13]. LCPD is characterized by lameness, pain, and limited movement of the hip, all of these symptoms are the result of avascular necrosis in FH [24–26].

According to the literature, the treatment and prognosis of the disease are determined according to the characteristics of each patient. Such as age, sex, type and size of the affected area in FH [27–31].

The limited information regarding the etiology of the disease represents the main question in LCPD. In this sense, and because multiple families have been described in different regions of the world, with more than one member affected. As well as various evidences have been considered, which indicate that there are inheritable genetic factors, which have an important role in the appearance of LCPD [51, 56–68].

On the other hand, avascularity around the FH plays a central role in the development of the disease. Alterations related to the vascular system, among these the triggers of prothrombotic states have been proposed by different authors. The proposed mechanism involves the formation of microthrombi that block blood flow in the vessels that supply the FH, resulting in avascularity. This theory has been related to different situations, such as the presence of FVL mutation and different thrombotic alterations in populations of patients suffering from LCPD. As well as the relationship with environmental factors, such as the link found between exposure to tobacco smoke and the development of LCPD [47–50].

On the other hand, inflammation could play a central role in bone destruction and remodeling, leading to the appearance of LCPD as well as more severe forms of the disease [65, 66].

It is important to point out that environmental factors such as malnutrition, obesity, mechanical overload and others previously exposed, will exacerbate the aforementioned mechanisms and the appearance of LCPD through different routes [14, 35–42].

## Conclusions

The pathogenesis LCPD is complex, so the degree of involvement is variable. Even though the etiology is unknown, the available information suggests that LCPD has a multifactorial etiology where multiple environmental, metabolic and genetic agents could be involved.

## Data Availability

Not applicable.

## Abbreviations

LCPD: Legg–Calvé–Perthes disease
FH: Femoral head
HDL: High density lipoproteins
TNF-α: Tumor necrosis factor alpha
IL: Interleukin
PT: Prothrombin
MTHFR: Methylenetetrahydrofolate reductase
eNOS: Endothelial nitric oxide synthase
